# Users with spinal cord injury experience of robotic Locomotor exoskeletons: a qualitative study of the benefits, limitations, and recommendations

**DOI:** 10.1186/s12984-020-00752-9

**Published:** 2020-09-11

**Authors:** Dominique Kinnett-Hopkins, Chaithanya K. Mummidisetty, Linda Ehrlich-Jones, Deborah Crown, Rachel A. Bond, Marc H. Applebaum, Arun Jayaraman, Catherine Furbish, Gail Forrest, Edelle Field-Fote, Allen W. Heinemann

**Affiliations:** 1 Northwestern University, Chicago, USA; 2 Shirley Ryan AbilityLab, Chicago, USA; 3 Shepherd Center, Atlanta, USA; 4 Kessler Foundation, East Hanover, USA; 5 Rutgers New Jersey Medical School, Newark, USA; 6 Division of Physical Therapy, Emory University, Atlanta, USA

**Keywords:** Outcome, rehabilitation, Focus groups, Assistive technology, locomotor training

## Abstract

**Background:**

Persons with spinal cord injury (SCI) may experience both psychological and physiological benefits from robotic locomotor exoskeleton use, and knowledgeable users may have valuable perspectives to inform future development. The objective of this study is to gain insight into the experiences, perspectives, concerns, and suggestions on the use of robotic locomotor exoskeletons by civilians and veterans living with SCI.

**Methods:**

Participants reported their demographic characteristics and the extent of robotic exoskeleton use in an online survey. Then, 28 experienced robotic locomotor exoskeleton users participated in focus groups held at three regional hospitals that specialize in rehabilitation for persons with SCI. We used a qualitative description approach analysis to analyze the data, and included thematic analysis.

**Results:**

Participants expressed that robotic exoskeletons were useful in therapy settings but, in their current form, were not practical for activities of daily living due to device limitations. Participants detailed the psychological benefits of being eye-level with their non-disabled peers and family members, and some reported physiologic improvements in areas such as bowel and bladder function. Participants detailed barriers of increased fatigue, spasticity, and spasms and expressed dissatisfaction with the devices due to an inability to use them independently and safely. Participants provided suggestions to manufacturers for technology improvements.

**Conclusions:**

The varied opinions and insights of robotic locomotor exoskeletons users with SCI add to our knowledge of device benefits and limitations.

## Background

Spinal cord injury (SCI) results in life-altering consequences in terms of morbidity, mortality, functional status, employment, and quality of life [1]. SCI prevalence is approximately 291,000 individuals in the United States, with nearly 17,730 new SCI cases each year [2]. SCI often results in reduced or complete loss of walking function and creates challenges with activities of daily living (ADL) [3, 4]. Persons with SCI may also experience secondary conditions, such as impairment of respiratory, cardiovascular, bladder, and bowel function, spasticity, pressure ulcers, osteoporosis and bone fractures, and chronic pain [5–8]. These functional limitations and secondary conditions often result in reduced community participation and quality of life [8].

Overground robotic exoskeletons can be used as rehabilitation tools and also as upright personal mobility devices. Regular use of robotic exoskeletons may help limit secondary conditions following SCI, including pain, spasticity, and decreased bone mineral density [9–11]; In addition, healthcare providers and home users acknowledge the psychological benefits that exoskeletons provide through eye-level social interactions and increased confidence [12, 13]. Therapists’ use of these devices is still in its infancy relative to user screening, therapy time, and dosage [14–17]. Studies describe user discomfort, lack of fall mitigation features, and limited utility as major design limitations [18]. The high cost of robotic exoskeletons also limits purchases by most potential users [15].

Current manufacturer requirements stipulate that home and community exoskeleton users must have a designated, trained companion during exoskeleton use. Selecting an efficient, consistent, and supportive companion to participate in training can be challenging [19]. Regardless of companion availability, many clinicians do not see community ambulation as a realistic goal for exoskeleton users given the current state of development of these devices, and instead prescribe them in home settings as a means of promoting upright health benefits and occasional completion of ADLs [15].

We need a better understanding of the experience of users to guide developments that enhance the efficiency, affordability, and usability of this technology. Comfort and safety are two major areas of concern not only for potential users, but also for therapists [20]. While there is a growing evidence base regarding the physical, social, and psychological benefits of robotic exoskeleton use, there is a limited evaluation of users’ experiences [21]. Thus, the aim of this study is to gain insight into the experiences, perspectives, concerns, and suggestions on the use of robotic exoskeletons by civilians and veterans living with SCI who have experience with these devices. This results of this report address the following four questions:

1. What are the experiences and perspectives of persons with SCI who have used a robotic exoskeleton?
2. How do persons with SCI appraise the benefits and barriers of robotic exoskeletons?
3. What concerns and limitations do persons with SCI perceive regarding robotic exoskeleton use?
4. What suggestions do experienced users with SCI have for robotic exoskeleton manufacturer consideration?

## Methods

Institutional review boards at collaborating sites approved the protocol. Participants provided informed consent and received an honorarium. The U.S. Army Medical Research and Development Command Office of Research Protections, Human Research Protection Office approved the protocol.

### Sample

This study used qualitative methods to address the aims by collecting survey data and conducting focus groups at three regional rehabilitation hospitals and referrals from Veterans Affairs (VA) hospitals where therapists provide specialized SCI care [1]: The Shirley Ryan AbilityLab (formerly the Rehabilitation Institute of Chicago) [2], Kessler Foundation [3], Shepherd Center, and [4] Jesse Brown and Minneapolis VA Hospitals. A moderator traveled to the facilities and facilitated focus groups at all sites with the exception of the VA Hospitals. Eligibility criteria were: SCI, age 18 years or older, able to read and speak in English, and experience using a wearable, overground robotic bi-lateral exoskeleton during rehabilitation or as a research participant.

### Procedures

Forty-eight adults completed a preliminary survey using REDCap [22] to report demographic information and to describe their experience with robotic exoskeletons. We attempted to recruit all survey participants for focus groups based on their availability and ability to travel to the local site. Research team members developed a focus group topic guide based on their research expertise and clinical experience (see Appendix). The moderator has 40 years of experience in designing and conducting qualitative research projects, and led focus groups while a court reporter took verbatim notes and provided a transcript. Focus groups were also audio-recorded. Personally identifying information was masked by a research team member who was uninvolved with coding to assure confidentiality. The Standards for Reporting Qualitative Research were used to guide manuscript preparation [23].

### Data analysis

We analyzed the interviews using qualitative description analysis with the intent to gain an accurate account of participants’ experience with the robotic exoskeleton [24]. We utilized the qualitative description approach as it allows for the analysis to stay closely connected to the participants’ words and events described, without significant interpretive inference. We imported the focus group transcripts and one individual interview transcript into QSR International’s NVivo 12 Pro software, reviewed the text, and confirmed that data were correctly imported and de-identified before analysis. We used a thematic approach to summarize participant responses [25, 26]. This qualitative approach involved a spiral analysis of open coding and interpreting the interviews line-by-line multiple times; reading and annotating the data; and describing, classifying, and interpreting the data into codes by three members of the research team. We used an inductive analytic approach to produce the codebook based on open coding of the first focus group interview [27]. Then, all authors met to review the first coded transcript, evaluate interpretations, reconcile discrepancies, discuss initial findings, make modifications, determine broad themes, discuss examples, and interpret themes. Next, different combinations of researchers coded the remaining transcripts with the codebook, including two primary coders who coded independently and then reconciled differences. A third coder read the transcript independently and reconciled the two primary coders’ themes. If coders did not agree, as indicated by kappa coefficients of less than 0.80, the team of three met to review codes and modify them to reach consensus. The codebook was refined throughout the coding process as more transcripts were analyzed by the research team. The research team created a master list of themes (e.g., participant experience, perspectives, recommendations), including interpretations that were categorized into superordinate themes (e.g., manufacturer limitations, research experience, emotional barriers). This list contained exemplary quotes from participants as well as diverging quotes. At this stage the results were described and a literature search of themes and superordinate theme categories was conducted to provide context to the results.

We used several strategies to assure methodological rigor. We enhanced interview reliability by using a standard, semi-structured topic guide and having the same moderator conduct all interviews. We assured investigator triangulation by having three investigators independently code transcripts before meeting to reconcile themes. Reconciled codes from the first two coders were confirmed independently by the third coder; they discussed discrepancies when kappa coefficients were below 0.80. Trustworthiness was ensured by having multiple independent coders. The entire research team met frequently to discuss the findings and analysis plans. We sought to increase credibility by creating a codebook early in the analytic process.

## Results

### Participant characteristics

Table 1 summarizes the participants’ demographic, injury, and experience characteristics. The sample consisted of 28 persons who were mostly male, white, middle-aged, and not working. Nearly two-thirds had sustained thoracic level injuries and over one-half had sustained incomplete injuries. The majority of participants gained robotic exoskeleton experience in research settings. The sample self-reported an average of two hours of robotic exoskeleton experience weekly over 12 months with a range of .5 months to 36 months after inpatient hospitalization and .5 h to 6 h per week.

**Table 1:** Sample Characteristics (*N* = 28)

Age	mean = 42 range 21–71
Time since injury (years)	mean = 9 range 2–28
Sex	
Female	32%
Race	
White	61%
Black	21%
Asian/Indian	7%
More than one	4%
Other	7%
Decline	0%
Hispanic/Latinx	
Yes	18%
Education Level	
9th–11th	4%
High School/GED	30%
Associates	7%
Bachelors	37%
Post-Baccalaureate	15%
Other	7%
Decline	0%
Occupational status	
Employed	26%
Student	15%
Unemployed	11%
Retired	30%
Other	18%
Decline	0%
Cause of injury	
Falls	18%
Vehicular Crash	29%
Violence	21%
Sport	21%
Pedestrian	4%
Other	7%
Injury Level	
Cervical	25%
Thoracic	64%
Lumbar	11%
Injury Severity	
Complete	36%
Incomplete	57%
Unknown	7%
Type of device used*	
Ekso	43%
Indego	43%
ReWalk	36%
Other	0%
Unknown	11%
Experience Type	
Rehabilitation Therapy	32.1%
Research	85.7%
At Home	3.6%
Outside of Your Home	7.1%

### Focus group themes

The results are described according to the four themes produced through our analysis: 1. experiences and perspectives, 2. benefits and barriers, 3. concerns and limitations, and 4. user suggestions. We summarize results by theme and provide representative quotes to illustrate the themes.

#### Theme 1: experiences and perspectives

The first topic area, “Experiences and Perspectives,” was related to the discussion of how participants engaged with robotic exoskeletons and their experience regarding the technology. Specifically, participants described how they became interested in robotic exoskeletons, the settings in which they were exposed to and used them, and their thoughts after using the device.

Participants indicated that they saw other individuals with SCI in the robotic exoskeleton and that encouraged them to try it themselves. Additionally, participants described interest in participating in research with the goal of walking.

*“I saw him doing it and I was like, oh, wait. I want to do that!”*

“*… just any way to help the advancement of helping individuals with spinal cord walk, I’m down. If I can be a part of that research, I want to be a part of it …*”.

Because of limited access, FDA requirements, and the purchase cost, participants said that the most experience they had with robotic exoskeleton was in a research setting.

*“The robot they use for outpatient is also used for research and then there’s a long list of people in outpatient that want to use it. Basically it’s like you can try it in outpatient, and then if you like it and you want to continue more than you can sign up for research.”*

Participants commented on the learning curve involved in learning to operate the robotic exoskeleton, indicating that it was difficult during the first few attempts but eventually was much easier to operate.

*“The first few sessions are really awkward -- just kind of learning the nuance of how that particular device moves and balance is a big thing, certainly for me based on the level of my injury and my core function. So staying upright is a challenge, especially at first. But after a few sessions you kind of get the feel of it.”*

*“I wasn’t as excited about my experience the first time, no. I would say it was just really rough and felt very -- like I had, as I said, robotic feeling. But my second time -- my second interaction with it was more fluid, so it was more positive. So I think over time it has gotten -- been a more encouraging outcome”.*

Many participants commented on how they had not had the ability to stand or walk for several years, so utilizing the robotic exoskeleton provided a unique opportunity to experience standing and walking.

*“I honestly just wanted to have the experience. Because it’s not every day you get to walk in a robot. You know, it’s like who wouldn’t want to do that? Especially from a person like in a wheelchair who hasn’t walked for years and just to get up and move around, show how tall I am and look over everybody, it was fun.”*

*“It’s pretty cool. It really was. So, all aspects of just being able to use it was very cool.”*

#### Theme 2: benefits and barriers

The second topic area, “Benefits and Barriers,” was related to the discussion of perceived personal benefits and barriers participants experienced using the device. In this section, benefits are discussed first and barriers follow.

Participants perceived several physiological, psychological, and social benefits associated with robotic exoskeleton use. Some participants reported improvement in bowel and bladder function, and pain management, as well as the opportunity for muscle stretching.

*“From the times that I’ve used it, if it was more than, I’d say, two days a week, it was definitely noticeable with bowel and bladder both.”*

*“Usually I walk in and my pain level is about a 6, 7, while I’m walking in the suit it’s gone as low as 2 to zero, which is great.”*

*“I enjoyed being able to feel the full stretch and extension of my legs in a step, in an actual step. It was -- it was nice to feel my body doing what it’s like made to do versus using just leg braces to walk”.*

One participant noted that the robotic exoskeleton was the most helpful modality regarding improving balance and mobility while other participants noticed improvements in mobility with continued use.

*“In my experience, it helped me the most. I’m able to balance longer, stand up longer, balancing longer, even walking up the stairs and standing there without assistance. And that all came from [the robotic] exoskeleton.”*

*“Immediately, I mean, I was able to take I think maybe 30-something steps the first day and I was like, wow, that’s great, and now we’re working our way up past like 1200 or something like that if it’s a good day.”*

Participants often mentioned the psychological benefits of utilizing robotic exoskeleton. Some participants expressed that the experience of walking provided them with the hope that one day they would be able to walk routinely.

“*… if you can especially see yourself walking, I think that’s a really like good thing for your mind to see you, again, taking those more natural gait steps, and like that positive feedback that it gives you hope.”*

*“Walking is a quality of life, you know? It’s standing. It’s exercising and just going through the motions of walking was great. Very encouraging to keep pushing to try to walk, even without the braces, so personally, I think it’s a great mental builder to my ability”.*

One of the most common benefits participants described was related to interacting with others at eye-level.

*“I just felt like I was mother again. And then when I got to look at my husband, and honestly I remember when I was over there with my husband and he kissed me just standing up, and he had tears in his eyes. And you just forget what it’s truly like because you can sit beside them on the couch or lay beside them in a bed, but when you’re going through the mall or somewhere, you’re rolling and they’re walking and it’s just -- it’s a different feeling. It makes you feel more complete so it helps mentally, too, not just physically”.*

*“The biggest thing for me is to be able to talk to somebody face to face standing up, because being in a chair it’s okay, you’re still communicating the same way, but to look them right in the eye as your talking to them is a big deal.”*

Although participants described many benefits from using the devices, some noted that they did not experience as many benefits as they initially expected.

*“There were certain benefits I was sort of expecting to get that I didn’t necessarily see. Like my feet still got swollen, I was hoping to see improvement. You know, I’m certain that, you know, the circulation was good, my blood pressure was good, but I still had swollen feet. My bowel [was] still really slow; that didn’t improve. So there is some things like physical things like that, that I was sort of anticipating a possible improvement with, within the 8 weeks, and granted it’s 8 weeks out of how many years of my body started developing whatever it is.”*

Participants noted several barriers to robotic exoskeleton use. Most notable were increased fatigue, the inability to utilize the device outside of research settings due to FDA qualification and research requirements as well as the experience of spasms.

*“But it’s very fatiguing, and then that fatigue kicks in a lot of spasticity.”*

*“I used it* [in a research study] *-- for me it was a great experience, but after the [research team] said I don’t qualify [to keep the device as I did not use the device frequently enough at home during the monitoring portion of the study].”*

*“When I was using [the robotic exoskeleton] I got a lot of spasms, and it was triggering that. I couldn’t really do it that much, my spasms kept kicking in”.*

#### Theme 3: concerns and limitations

The third topic area, “Concerns and Limitations,” relates to limitations of the device technology compared to an ideal robotic exoskeleton. Participants discussed safety and social concerns of using the device and practical limitations of the technology, including fall risk and pressure ulcers, and risk of skin compromise.

*“There are times that you could really misstep on it and you have no one with you and they have to catch it from the back. If not I would be falling with it face down. It’s either face down or backwards, and there’s no stopping it because it’s the robot.”*

*“I don’t think I had ever felt safe using it outside, in an -- in sort of an uncontrolled setting because if I fell, I’d be … out of luck; excuse my expression. The thought of breaking a bone.”*

Participants described the requirement of having a companion present in order to use the device as the biggest limitation.

*“You are not going to be able to use it by yourself at home. You cannot even get into the car with it because it’s too big, clunky. How are you going to sit with it with the battery on your back?”*

Some users stated that the public perception of someone using a robotic exoskeleton is no different from that of someone using a wheelchair, while others expressed frustration or concern about being vulnerable or stigmatized while using an exoskeleton. Some participants described frustration that able-bodied individuals expect robotic exoskeletons to be of great value, but because of the limited benefits, persons with SCI may not find them of the same value as able-bodied individuals.

*“With people saying: Well, don’t you wish you had that all of the time? My answer is: Well, not really, that exoskeleton is not going to let me care for my daughter or go to work or get in my car or do all of the things that I need to do during the day. And so in some part I was a little frustrated that people focus so much on: Oh, yeah, you got to stand. When of all of the things in my life, that is not the most frustrating or even pressing issue.”*

Other concerns included bulky robotic exoskeleton and the amount of time required to don and doff the device, risk of falls, slow gait speed, spasticity limiting device use, difficulty transferring in and out of an automobile, and limited feasibility in a work environment. The cost to purchase an exoskeleton was also a concern.

*“Other than it’s too hot, too expensive. Can’t afford it.”*

“*It costs as much as an investment property.”*

Participants discussed the contrast between their desired outcomes of using the device compared to the reality of using the device. Using a robotic exoskeleton requires both hands to balance and stand upright, limiting the participant from simultaneous activities that require use of both hands.

*“Even if you could fit into a car with it, are you going to go to the grocery store and take an hour to go down aisle one to grab a jug of milk and come back?”*

*“It requires you use both hands. So, you can’t do too many other things but walking or stand, that’s it.”*

Although participants did not find practical value in the device in their everyday world, they thought that the robotic exoskeleton was of great utility in therapy.

*“I love the thing as far as therapy, I’m there 100 percent, but my everyday life I couldn’t use it.”*

#### Theme 4: user suggestions

The fourth topic area, “User Suggestions,” describes the ways participants hoped that robotic exoskeletons could be improved to address their concerns.

Participants suggested various items or functions to device manufacturers, of which greater mobility, faster speed, lighter weight, and ability to walk up and downstairs were mentioned frequently. Participants wanted use of both hands in order to be more functional and productive while using the exoskeleton. Participants would like the devices to incorporate additional actuators to promote balance and core function, perhaps with gyroscopes. Clothing-like features with ability to don and doff quickly were among the most desired features.

*“Speed and mobility, accessibility. Like being able to put it on without assistance. Some type of anti-fall resistant mechanism type of thing where it can actually catch you and give you a chance to not fall over.”*

*“It better be able to go upstairs. I better be able to put it on by myself in five minutes. I better be able to use it by myself. Like you mentioned fall prevention, so if it incorporated like a gyroscope or something that would be a mechanism that would allow it to stop you from falling, self-balance in a way...And it’s got to be small enough that I can still wear it in my wheelchair if I need the chair. And get in and out of my car with it on. So, that’s a lot, but for 50 grand, it’s got to be functional.”*

Users suggested other features to improve accessibility and ease of use through greater adjustability, less obtrusive size, and capacity for transfers from wheelchair to robotic exoskeleton and in and out of cars. Finally, participants recommended that robotic exoskeletons have greater functionality by adding intuitive features like integrated functional muscle stimulation to activate muscles.

*“Something that you can wear while you’re in your wheelchair. You can stand up … walk around the room or whatever and not so much just wear it all day … with battery, you got to have some kind of battery pack, but something that you can wear in the chair, stand up, walk around the classroom, do what you got to do, sit back down, go somewhere else, and do the same thing.”*

*“If they made it where it turns into like a chair where you could transfer to the car. And, you know, it lowers, you transfer to the car, and you can transfer back to it, and up.”*

## Discussion

We sought to understand better the experiences of persons with SCI who have used robotic exoskeletons. Focus group participants’ comments reflected a mix of hope regarding the future of robotic exoskeleton technology and of reality regarding their immediate utility as a therapy aid. Most participants were intrigued and enthusiastic about the prospect of improving or regaining the ability to walk, though their enthusiasm was tempered by the constraints of the technology and access to it. Of note, over half of the sample had an incomplete spinal injury, which is associated with greater likelihood of improving or regaining the ability to walk than with a complete injury. Participants indicated that purchasing this technology for personal use was not feasible given the cost; thus their access to robotic exoskeletons was typically through a research project or in a clinical setting, consistent with the research literature [13, 19, 28].

Companion requirements to assist with donning and doffing the device and to assure safety while walking precluded independent use. Bryce et al., suggests that individuals should be able to don and doff an exoskeleton independently in five minutes or less for it to be considered viable for community use; however, an 8-week research study evaluating donning and doffing a robotic exoskeleton after 24 training sessions with 32 participants indicated that although 84% of the sample could don and doff the device, the average time was 9:01 and 2:44, respectively [29–31]. The complexity of donning and doffing and limited feasibility in community settings is reflected in our data. Users identified other practical constraints, including inability to carry items while using the exoskeleton and limited ability to perform ADLs, walk on uneven surfaces, climb stairs, transfer, and sit comfortably. Exoskeleton users also described as limitations the inability to walk at a self-selected pace and the risk of falls. These considerations limit its use as a routine mobility aid. Users also described minor discomforts such as skin irritation and fatigue which have been reported by other investigators [31]. However, while users did not perceive current robotic exoskeletons as a practical mobility solution, some participants emphasized the therapeutic value of robotic exoskeletons as a means for improving upright posture, balance, and mobility.

In addition to mobility, users cited the health benefits resulting from upright posture and movement as a reason to use an exoskeleton [11]. Users reported mixed experiences in this regard. There was a range of experiences with some declaring the devices as helpful in improving bowel and bladder function, while others did not. In contrast, users cited psychological and social benefits universally. The experience of standing, in and of itself, and being at eye level with others, facilitated social interactions and enhanced self-confidence, consistent with prior studies [17, 18].

This study has several design features that limit the reproducibility of the findings. The results only reflect the experience of participants recruited from the three centers and may not represent the robotic exoskeleton experience of all persons with SCI or other impairments. The results also do not reflect the amount of time spent in the device and therefore may affect physiological changes. Despite multiple and sustained efforts, we were not able to recruit a sufficient number of veterans for a focus group, so we conducted semi-structured interviews. The interview guide detailed the probing questions related to the potential benefits that persons with SCI may have experienced when using the robotic exoskeleton, but did not explicitly state all probing questions regarding the potential issues experienced by participants. Although the moderator thoroughly inquired about the issues participants experienced, not having the probing questions directly in the semi-structured interview guide may have resulted in a bias toward more description of the benefits experienced. Although we tried to establish a non-evaluative climate, the focus group format may have limited participants’ willingness to voice opinions that conflicted with the perspectives of others. Nonetheless, we reached thematic saturation after the third focus group; the additional group and interviews did not yield more information, consistent with the suggested three to five focus groups typically needed for qualitative research [32].

Future studies should seek to evaluate the physiological, functional, social, and psychological benefits of robotic exoskeleton in clinical and community samples. Clinical research should focus not only on participants’ health outcomes, but also on integrating technology into gait therapy in inpatient and outpatient settings. Meanwhile, robotic exoskeletal technology continues to develop and these developments may improve users’ experience and allow greater deployment.

## Conclusion

Participants described a range of benefits to robotic exoskeleton use including psychological benefits of eye level contact and physiological benefits of balance, gait, and improved bowel and bladder function. Participants also described many barriers they encountered when using the robotic exoskeleton such as increased fatigue, spasticity and spasms, and not fulfilling their expectations of physical improvements. Regarding device limitations of robotic exoskeletons, participants described safety concerns due to fall risk, inability to use the device independently, and the inability to complete daily activities. Participants stated that robotic exoskeletons were useful as a rehabilitation tool, but they perceived the current technology as not being suitable and affordable for home and community use.

## Data Availability

Audio files and transcripts are not available for review because of the risk of participant identification.

## Appendix

**Table 2:** Focus Group Guide

1. Tell me about your experience using a robotic exoskeleton, devices that help people with spinal cord injury walk. a. Why did you/do you choose to use a robotic exoskeleton? b. Who did you talk to? c. What kind of support did you have? d. What went into your decision-making experience?	
2. Show video clips of several robotic exoskeletons. Add information regarding donning and doffing, weight of exoskeleton, batteries, of each of the types of exoskeletons. (Still shot of each device in the community). (Check You Tube videos)	
3. Tell us your experiences with peer mentors or formal peer support regarding the use of exoskeletons.	
4. How have your previous experiences with technology influenced your thoughts on exoskeletons?	
**Therapists can use these devices to help people walk, exercise, train mobility, and other health care benefits after their spinal cord injury.**	
5. How have you experienced robotic exoskeletons in therapy? a. What activities did you do with the device? b. What benefits did you experience? c. What, if any, risks did you experience? d. What are the device limitations you experienced?	
6. Some people use robotic exoskeletons to help get around their home and neighborhood. a. How have you experienced robotic exoskeletons **in your home**? b. How have you experienced robotic exoskeletons **in your neighborhood**? Doing what?	
7. How have you benefitted from using the robotic exoskeleton? a. Tell me about the physical benefits Probes: i. What cardiovascular benefits? ii. What spasticity benefits? iii. What skin integrity benefits? iv. What changes in pain occur? v. What bladder and bowel benefits? vi. What strength/endurance benefits do you see? vii. What weight benefits do you see? b. What about social benefits? Probes: i. What benefits to recreational activities? ii. What benefits to community participation? iii. What benefits for family interactions? iv. What benefits for life satisfaction? v. What benefits for independence? c. What about occupational benefits? Probes: i. What benefits for ADL’s? ii. What benefits for work outside the home? iii. What benefits for work inside the home? iv. What benefits for further education and/or occupational goals? v. What benefits for self-care? d. What about emotional benefits? Probes i. What benefits to self-confidence? ii. What benefits to resilience? iii. What benefits to anxiety and depression? iv. What changes to perceived stigma? v. What changes to grief/loss? e. What other benefits?	
8. What issues have you experienced with these devices? [Probe for safety, economic, functional issues] a. Tell me about the physical issues. b. What about social issues? c. What about occupational issues? d. What about emotional issues? e. What other issues?	
9. How do you think your family, friends, or the public perceive your use of an exoskeleton?	
10. What has been your reasoning for purchasing, or not purchasing, a robotic exoskeleton? a. [If purchased] May I ask what and how you paid for the exoskeleton? b. How do you perceive the value you get, or would get, for your purchase? c. Would it be worth it to buy or lease one for your own use? [Why or why not?]	
11. What advice would you offer potential users of robotic exoskeletons?	
12. What improvements would you like to see in robotic exoskeletons? a. What are the limitations of available models? b. What features would you like to see changed or developed in new devices?	
13. What other aspects of exoskeleton use that we have not discussed would you like to mention?	

## Abbreviations

SCI: Spinal Cord Injury
ADL: Activity of Daily Living
VA: Veterans Affairs
